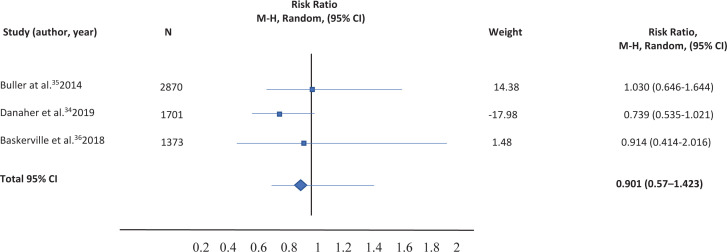# Corrigendum: Effectiveness of mobile applications to quit smoking: Systematic review and meta-analysis

**DOI:** 10.18332/tpc/135167

**Published:** 2021-04-21

**Authors:** Raquel Cobos-Campos, Arantza Sáez de Lafuente, Antxon Apiñaniz, Naiara Parraza, Iraida Pérez Llanos, Gorka Orive

**Affiliations:** 1 Bioaraba Health Research Institute, Epidemiology and Public Health research group, Vitoria-Gasteiz, Spain; 2 Osakidetza Basque Health Service, Lakuabizkarra Health Centre, Vitoria-Gasteiz, Spain; 3 Osakidetza Basque Health Service, Olaguibel Health Centre, Vitoria-Gasteiz, Spain; 4 School of Pharmacy, Laboratory of Pharmaceutics, University of the Basque Country UPV/EHU, Vitoria-Gasteiz, Spain; 5 Bioaraba Health Research Institute, Nanobiocel research group, Vitoria-Gasteiz, Spain; 6 University Institute for Regenerative Medicine and Oral Implantology, Foundation Eduardo Anitua, Vitoria-Gasteiz, Spain; 7 Singapore Eye Research Institute, Singapore, Singapore

**Keywords:** smoking cessation, mobile applications, MeSH Unique ID: D063731, Telemedicine MeSH Unique ID: D017216

**Corrigendum on:**

**Effectiveness of mobile applications to quit smoking: Systematic review and meta-analysis**

**By Raquel Cobos-Campos, Arantza Sáez de Lafuente, Antxon Apiñaniz, Naiara Parraza, Iraida Pérez Llanos, Gorka Orive**

**Tobacco Prevention and Cessation, Volume 6, Issue November, Pages 1-11**

**Publish date: 10 November 2020**

**DOI: https://doi.org/10.18332/tpc/127770**

An error in data entry occurred during the production tables 2 and 3 in the manuscript, as the authors accidentally omitted the confidence interval dashes in the tables.

Table 2. Comparison of smartphone app versus other intervention (routine practice, text messaging, app for computer or tablet)

The table is the following:

**Figure:**
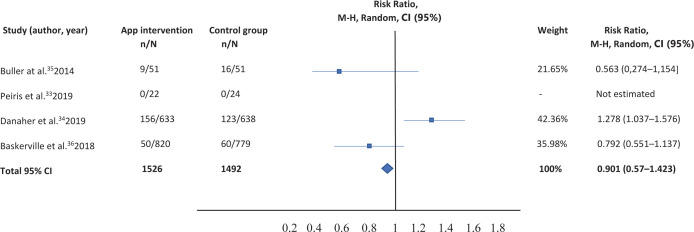


The correct table should be:

**Figure:**
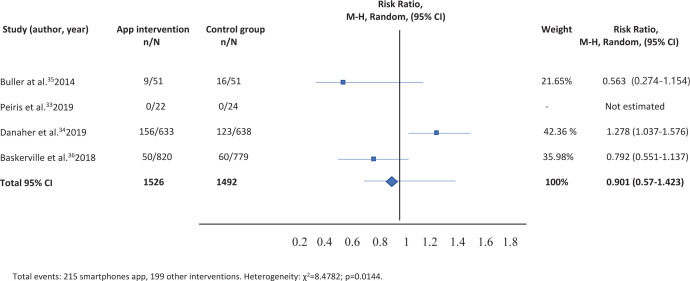


Table 3. Comparison of smartphone app versus other intervention: Sensitivity analysis

The table is the following:

**Figure:**
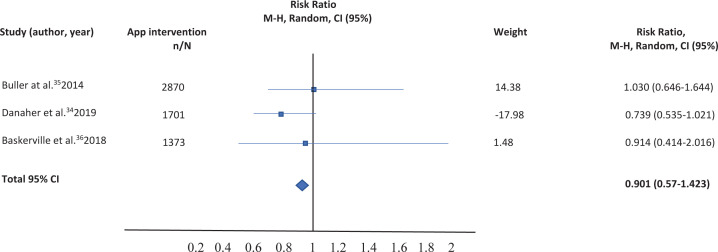


The correct table should be:

**Figure:**